# A transformer model for cause-specific hazard prediction

**DOI:** 10.1186/s12859-024-05799-2

**Published:** 2024-05-03

**Authors:** Matthieu Oliver, Nicolas Allou, Marjolaine Devineau, Jèrôme Allyn, Cyril Ferdynus

**Affiliations:** 1 Methodological Support Unit, Reunion University Hospital, Saint-Denis, La Réunion France; 2 Clinical Informatics Department, Reunion University Hospital, Saint-Denis, La Réunion France; 3 Intensive Care Unit, Reunion University Hospital, Saint-Denis, La Réunion France; 4 Clinical Research Department, INSERM CIC1410, Saint-Pierre, La Réunion France

**Keywords:** Transformer, Competing risks, Cause-specific hazard, Synthetic data, English longitudinal study of ageing

## Abstract

**Backgroud:**

Modelling discrete-time cause-specific hazards in the presence of competing events and non-proportional hazards is a challenging task in many domains. Survival analysis in longitudinal cohorts often requires such models; notably when the data is gathered at discrete points in time and the predicted events display complex dynamics. Current models often rely on strong assumptions of proportional hazards, that is rarely verified in practice; or do not handle sequential data in a meaningful way. This study proposes a Transformer architecture for the prediction of cause-specific hazards in discrete-time competing risks. Contrary to Multilayer perceptrons that were already used for this task (DeepHit), the Transformer architecture is especially suited for handling complex relationships in sequential data, having displayed state-of-the-art performance in numerous tasks with few underlying assumptions on the task at hand.

**Results:**

Using synthetic datasets of 2000–50,000 patients, we showed that our Transformer model surpassed the CoxPH, PyDTS, and DeepHit models for the prediction of cause-specific hazard, especially when the proportional assumption did not hold. The error along simulated time outlined the ability of our model to anticipate the evolution of cause-specific hazards at later time steps where few events are observed. It was also superior to current models for prediction of dementia and other psychiatric conditions in the *English longitudinal study of ageing* cohort using the integrated brier score and the time-dependent concordance index. We also displayed the explainability of our model’s prediction using the integrated gradients method.

**Conclusions:**

Our model provided state-of-the-art prediction of cause-specific hazards, without adopting prior parametric assumptions on the hazard rates. It outperformed other models in non-proportional hazards settings for both the synthetic dataset and the longitudinal cohort study. We also observed that basic models such as CoxPH were more suited to extremely simple settings than deep learning models. Our model is therefore especially suited for survival analysis on longitudinal cohorts with complex dynamics of the covariate-to-outcome relationship, which are common in clinical practice. The integrated gradients provided the importance scores of input variables, which indicated variables guiding the model in its prediction. This model is ready to be utilized for time-to-event prediction in longitudinal cohorts.

## Introduction

Survival analysis under competing risks describes the time of occurrence of the first of several possible outcomes. This can be done by predicting the cause-specific hazards from a set of explanatory variables, also called covariates. Competing risks have countless applications in a system’s failure time, which includes client churn and probability of a borrower defaulting on a loan [1, 2]. In medicine, modelling competing events can be used to measure the time-to-event on several possible outcomes such as treatment effects on a patient or the prediction of the time of death after colon cancer diagnosis [3, 4].

Previous work was done on the prediction of cause-specific hazards under competing risks. Firstly, the semi-parametric Cox proportional hazards (CoxPH) model was introduced for survival analysis under the assumption of proportional hazards, namely a linear relationship between the log-hazard ratio and the covariates [5]. Because the original CoxPH model failed in the context of variable collinearity when applied to highly dimensional data, the Regularized CoxPH (RCoxPH) was introduced. This model minimizes CoxPH’s partial likelihood function with an additional elastic net penality [6]. This model had numerous uses, such as the identification of breast cancer prognosis markers [7]. Secondly, a collaspsed log-likelihood approach was developed and applied to colon cancer data [4]. This method does not rely on the proportional hazards assumptions of the CoxPH model, which improved its applicability to real-world data. It was recently implemented as a Python package in PyDTS [8]. Lastly, several studies used deep learning models to minimize a loss function adapted to datasets with censored data [9]. Multi layer perceptron models outperformed previous models in both continuous (DeepSurv) and discrete time (DeepHit) [10, 11]. These deep learning models are able to learn without strong assumptions on the predicted hazard rates; however, they were not initially designed to handle temporal covariates or produce temporal predictions, which limits their performance in survival analysis on longitudinal cohorts.

Additionally, several studies reported on the failure of the proportional hazard assumption in survival analysis, notably for treatment response and oncology [12–15]. This highlights the need for modelling competing risks with non-proportional hazards.

In various tasks involving sequential data, such as natural language processing and time series forecasting, Transformer-based models demonstrated excellent performance in learning complex dynamics from sequential data [16, 17]. Transformer models are particularly suited for sequence generation, which motivated their use in time series predictions of discrete time cause-specific hazards. A Transformer model was recently used for survival analysis with a single event [18]. In this study, we introduce a Transformer-based deep learning model for the prediction of the cause-specific hazards in discrete time under competing risks.

Because the true data-generating mechanisms that entail targeted cause-specific hazards are unknown in practice, we used synthetic data to compare our model against three state-of-the-art models [19]. We followed the ADEMP guidelines (Aims, Data-generating mechanisms, Estimands, Methods, and Performance Measures) for simulation and reporting of results [20]. We then validated our model on the English longitudinal study of ageing (ELSA) dataset for the prediction of *death*, *dementia* and *psychiatric conditions* [21]. To our knowledge, this is the first study to use a Transformer-based model for the prediction of the cause-specific hazards in discrete-time under competing risks.

This article is organized as follows: in “Methodology” section describes our Transformer-based model, the benchmark models, as well as the simulated and ELSA datasets; in “Discussion” section presents the predictive performance of each model on the synthetic and ELSA datasets; finally in “Conclusions” section, we discuss the present conclusions of this study.

Our codes are openly available at https://github.com/USM-CHU-FGuyon/cause_specific_hazard_transformer.

## Methodology

### Notations

Competing risks analysis considers a patient described by a vector of covariates *X*, that may experience one of *E* separate events on a [0, *T*] period of time. A patient may be censored at $t_0 \le T$, in which case it is only known that no event occurred before $t_0$. For convenience, competing events were denoted $\{1, \dots , E\}$. If event *e* occurred at time *t*, the outcome is written (*e*, *t*) with $e \in \{0, 1, \dots , E\}$, $t \in [0, T]$, and $e=0$ indicating *censoring*.

The cause-specific hazard $\lambda _{e, X}(t)$, for $e\ge 1$, defined by (1) is the instantaneous rate of occurrence of event *e* at time *t*, given that the patient remained event-free until *t*. A model of cause-specific hazard explores the relation between covariates *X* and the cause-specific hazard $\lambda _{e, X}$ for each event *e* [22].

1 $$\begin{aligned} \lambda _{e, X}(t) = \lim _{\delta \rightarrow 0}\frac{P_{e, X}(t \le T < t+ \delta \mid T > t)}{\delta } \end{aligned}$$

Note that in discrete-time competing risks, the cause-specific hazard is defined as a probability and not as an unbounded positive number [23]. We also introduce the cumulative incidence function (2). This is a function of the cause-specific hazard that describes the proportion of patients that experienced event *e* up until time *t*.

2 $$\begin{aligned} I_{e, X}(t) = \sum _{\tau = 0}^{t} i_{e, X}(\tau ) \end{aligned}$$

where $i_{e, X}$ is the incidence function defined by:

3 $$\begin{aligned} i_{e, X}(\tau ) = \lambda _{e,X}(\tau ) \prod _{k = 0}^\tau \left( 1 - \sum _{e \in \{1, \dots , E\}} \lambda _{e,X}(k)\right) \end{aligned}$$

The goal of this study is to build a prediction model for the cause-specific hazards $(\lambda _{e, X})_{e \in \{1, \ldots , E\}}$ from a set of covariates *X*. This study focused on the cause-specific hazard but did not explore the prediction of the sub-distribution hazard. In the following, *X* may be constant or longitudinal data.

### A transformer-based model for cause-specific hazard prediction in discrete time

We used a Transformer-based deep learning model to predict the cause-specific hazard $\lambda _{e, X}$ of each event *e* from covariates *X*. This section describes the input and output data, the loss function that was minimized and the model architecture.

#### Input and output data

In real-world applications, the cause-specific hazards are unknown. The available data are the covariates *X* and outcomes (*e*, *t*) where *e* is the experienced event—or censoring—and *t* the time-to-event. Our model predicts the cause-specific hazards $\lambda _{e, X}$ of events *e* from the covariates *X* as a time series of length *T*. The output of the model may be written as matrix (4).

4 $$\begin{aligned} \lambda _X = \begin{bmatrix} \lambda _{1,1}&{}\quad \ldots &{}\quad \lambda _{1,T}\\ \vdots &{}\quad \ddots &{}\quad \vdots \\ \lambda _{E,1}&{}\quad \ldots &{}\quad \lambda _{E,T}\\ \end{bmatrix}_{E \times T} \end{aligned}$$

#### Loss function

The collapsed log-likelihood (5) from the PyDTS package was used as a loss function [8]. This function evaluates the consistency between the predicted cause-specific hazards $\lambda _{X=x}$ and the observed outcome $(e_x,t_x)$.

5 $$\begin{aligned} L(\lambda _{X=x}, e_x, t_x) = \sum _{j = 1}^E\sum _{k = 0}^{t} \delta _{jk}^{et} \log {\lambda _{j,k}(x)} +\left( 1- \delta _{jk}^{et}\right) \log {(1-\lambda _{j,k}(x))} \end{aligned}$$

where

$$\begin{aligned} \delta _{jk}^{et} = 1 \text{ if } (j,k) = (e_x, t_x) \text{ else } 0 \end{aligned}$$

Minimizing this loss encourages:

- A high value of $\lambda _{e,t}(x)$; which represents the predicted hazard for the observed outcome $(e_x, t_x)$
- Low values of $\lambda _{j,k}(x)$ for $(j,k) \ne (e_x,t_x)$; which represent the predicted hazard for outcomes that were not observed

Note that a patient censored at $t_{x}$ will contribute to low values of $\lambda _{j,k}(x)$ for each event *j* and each time $k < t_{x}$.

#### Transformer-based model architecture

The Transformer model is a sequence-to-sequence architecture that was introduced as a response to the vanishing-gradients problem that faced long short-term memory (LSTM) and other recurrent neural networks [24]. It utilizes the self-attention mechanism in an encoder–decoder architecture to learn complex temporal features of input and/or output data. They are especially suited for producing meaningful sequential output, which initially motivated their use for NLP tasks. A gentle introduction to the Transformer architecture is provided in Appendix 1. Consequently, the Transformer architecture also proved to be efficient for time series prediction from sequential or constant input data.

**Fig. 1:**
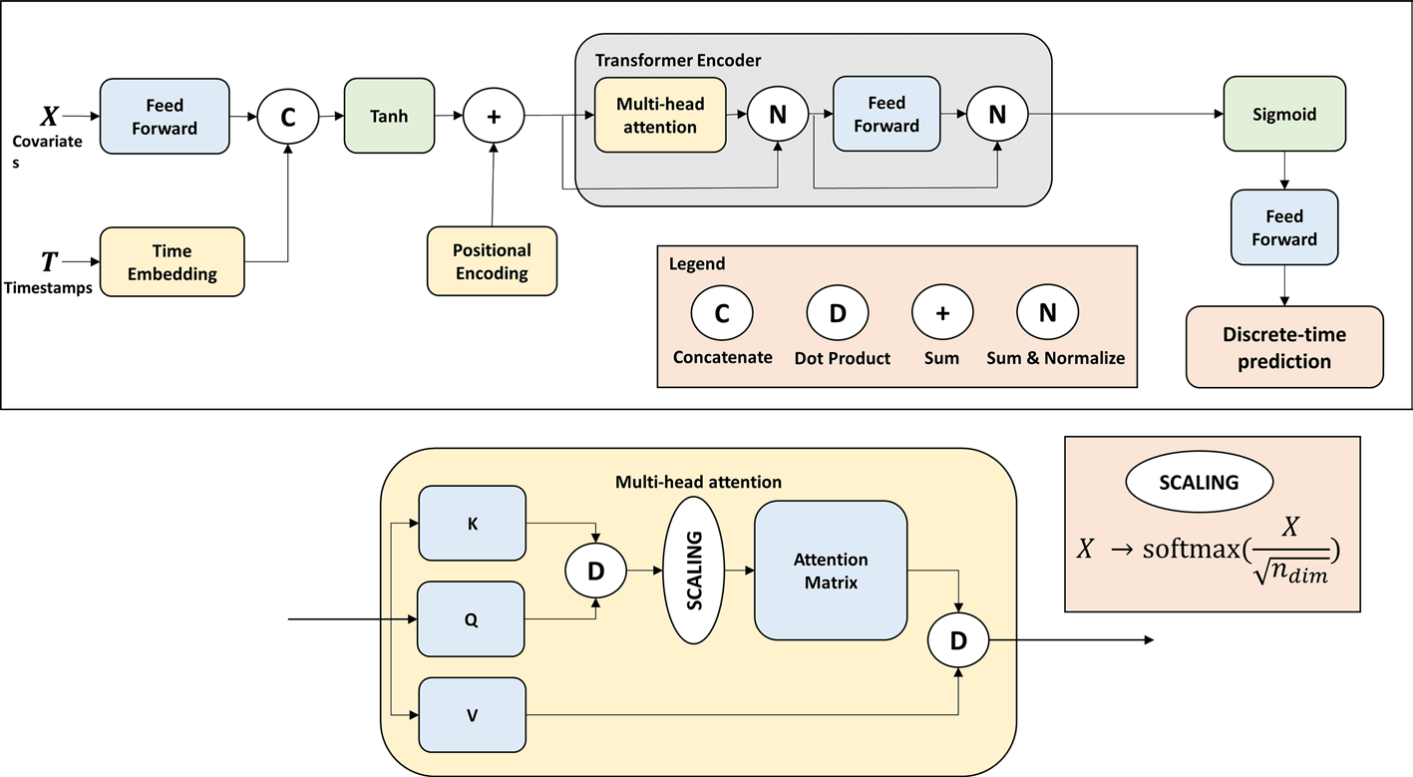
Architecture of our transformer-based model. Each part of the architecture is described in detail in “Appendix 1”

Our model architecture is presented in Fig. 1. It is based on a Transformer encoder, and a linear decoder to predict cause-specific hazards as a time series for each event. An input vector of covariates *X* is encoded by a linear layer and concatenated with an embedding of time. A positional encoding is summed to the obtained tensor, and fed to the Transformer encoder that outputs a single time series of length $E \times T$. This time series is then decoded into a matrix of shape (*E*, *T*) by a single linear layer. The loss function (5) ensures that the model learns to predict cause-specific hazards. This model was implemented using the Pytorch framework.

### Performance evaluation

#### Benchmark models

The performance of our Transformer-based model in predicting cause-specific hazards was compared to three existing models.

Firstly, we used the semi-parametric RCoxPH model from the *lifelines* package in Python [25]. Secondly, we used the PyDTS model from Lee and al. [4, 8]. Finally, we implemented a model equivalent to the original DeepHit model using the Pytorch framework [11]. This contains a feed forward subnetwork with one hidden linear layer for each competing event and minimizes the loss function (5). All models predicted a time-discretized cause-specific hazard for each competing event in the form of a $E\times T$ matrix, as presented in (4).

#### Benchmark designs

We evaluated all models using the same experimental setup, for both the synthetic and ELSA data. Data was split as 80% for training and 20% for validation. As described in “Loss function” section, models learned to predict patients’ cause-specific hazard for each competing event by learning from observed events in the training data. Both deep learning models had 64-neurons hidden layers and no dropout.

Additional implementation details are available in our code repository.

***Synthetic data benchmark***

**Fig. 2:**
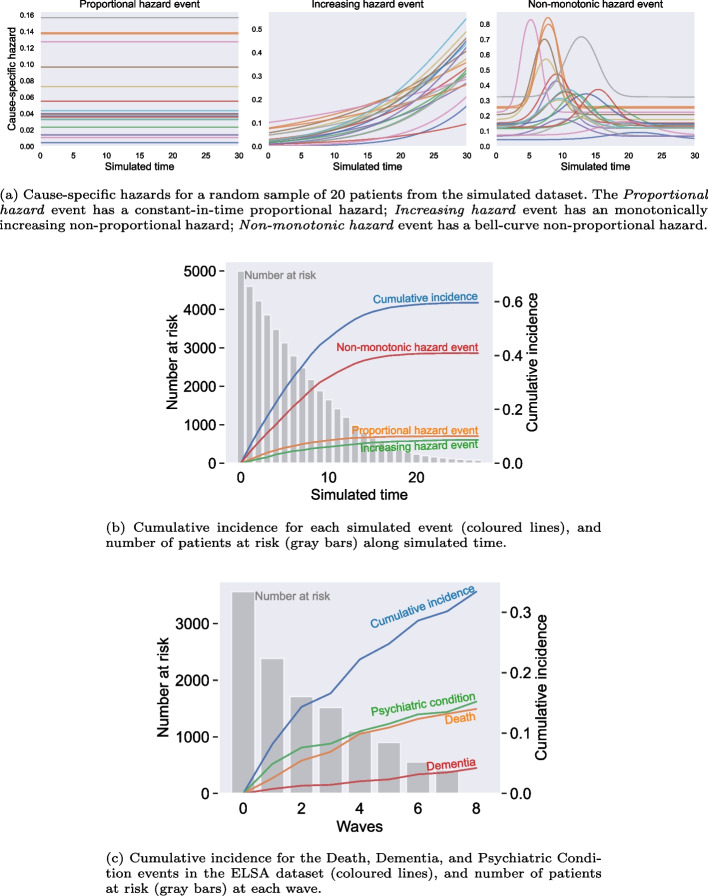
Description this study’s data. **a** and **b** respectively illustrate underlying cause-specific hazards and the cumulative incidence of each simulated event. **c** illustrates the cumulative incidence function of events in the ELSA cohort

We simulated populations of 2000—50,000 patients described by five covariates and susceptible to experience three competing events. Their covariates were independent and uniformly distributed between 0 and 1. Events were drawn using cause-specific hazard functions defined in Table 5 from Appendix. Cumulative incidences of each event, and the number of patients at risk at each time step are illustrated in Fig. 2a. Note that one of the simulated events’ hazard was proportional and the other two were non-proportional. Departure from proportional hazard hypothesis is common in clinical data, but represents a strong limitation for most survival analysis models [12].

Finally, censoring times were drawn uniformly between 1 and 49. A patient was censored if the drawn censoring time was anterior to the drawn event. Events (and censoring) were drawn 10 times separately, training and evaluation were done on each drawn dataset to measure performance variability.

In this synthetic experiment, ground truth cause-specific hazards are known. For this reason, model predictions were evaluated on the mean absolute error of the cause-specific hazard prediction. We also evaluated the models’ predictive performance along simulated time, and with varying training sample size.

***ELSA data benchmark*** The ELSA dataset is a representative cohort of the English population older than 50. It features economic, social, psychological, cognitive, health, biological and genetic data [21]. This longitudinal study currently features 9 waves of data acquired over 18 years and includes various diagnoses of cardiovascular, ocular, and psychiatric diseases.

We used this longitudinal cohort to evaluate the models’ prediction of *dementia* and *psychiatric conditions*. The ELSA dataset refers to a psychiatric condition for any of the following psychiatric disorders: hallucinations, anxiety, depression, emotional problems, schizophrenia, psychosis, mood swings, and manic depression. Our study population was the cohort from wave 2 that started in 2004. Patients already diagnosed for a psychiatric condition or dementia were excluded. Because mortality data was last updated in 2012, the study period was 2004–2012. We evaluated the models on the following competing events:

- *Dementia* new diagnosis of dementia
- *Psychiatric condition* new diagnosis of a psychiatric condition
- *Death*

Contrary to our synthetic dataset, the ground-truth for the cause-specific hazard is unknown; hence, models were evaluated on the Integrated Brier Score and Time-dependent Concordance Index for each event [26, 27]. The Brier Score is a generalization of the mean absolute error applied to the comparison of predicted probabilities and observed event. The Concordance Index is a generalization of the area under receiver operating characteristic (AUROC), it evaluates the ranking of failure times from the predicted probabilities [28]. The Integrated Brier Score and Time-dependent concordance index are respective variants of the brier score and concordance index adapted to the prediction of time series. The mean error and $95\%$ confidence intervals were computed by bootstrapping on the test dataset. Finally, the assumption of proportional hazards was evaluated by computing the *p* values of the Schoenfeld residuals from the RCoxPH model [29].

We used the Integrated Gradients method on both deep learning models to provide an importance score for the input features [30]. This method provides importance scores with a lower computational cost than Shapley values when applied with a large number of input variables and time series output. In this work, we present the total importance scores over the whole ELSA dataset; however, these scores are available at each prediction. Such importance scores were shown to improve to the usability of artificial intelligence in clinical practice [31].

## Results

### Evaluation on synthetic data

#### Simulated data

We simulated datasets of sample sizes of 2000, 5000, 10,000, 20,000, and 50,000 patients each described by 5 covariates and susceptible to experience one of 3 competing events during a period of 30 timesteps. In total, approximately $40\%$ of patients were censored.

A sample of simulated cause-specific hazards for each event are shown on Fig. 2a. We introduced three simulated events: a *Proportional hazard* event that had constant hazard in time, and two *non-proportional hazard* events: denoted the *Increasing hazard* and *Non-monotonic hazard* events which featured a temporal evolution with a non-linear dependence on the covariates. The *Non-monotonic hazard* event had a bell-curve distribution where parameters of mean and standard deviation depended on patients’ covariates (see Table 5 from Appendix).

Figure 2b shows the cumulative incidence of each of the three events over the simulated time. We noted that fewer events were observed at the later timesteps of the simulated time due to a smaller number at risk.

#### Performance comparison

**Fig. 3:**
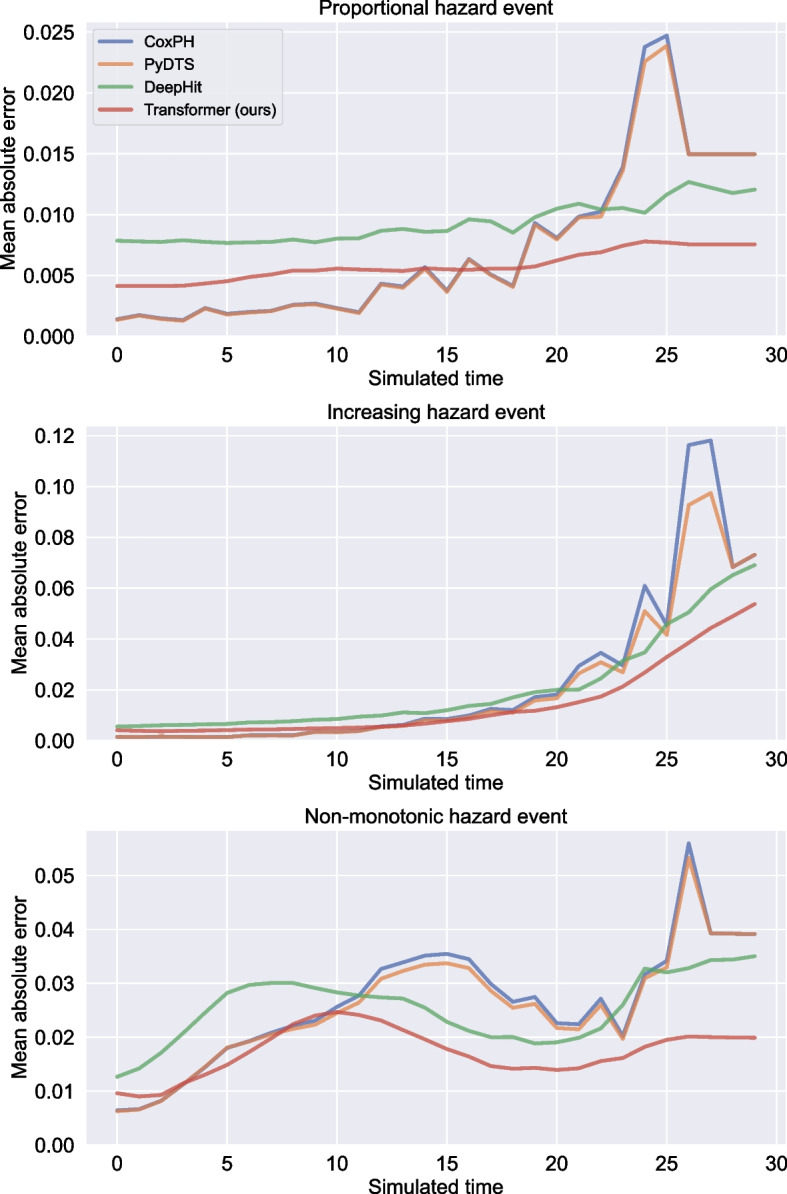
Time-dependance of the models’ performance. Performance was computed using the mean absolute error for the prediction of the cause-specific hazard for each simulated event. The Transformer model surpassed other models by a large margin on non-proportional hazard events, thanks especially to a major performance gap on the second half of the simulated time. It was also better than the DeepHit model at every single time step. This error was computed with each models being trained on a dataset of 10,000 simulated patients

The mean absolute error of the cause-specific hazard prediction for several sizes of synthetic datasets is presented in Table 1. The Transformer-based model outperformed or equalled other models on non-proportional hazard events for all dataset sizes, and was better or equivalent to other models on the *Proportional hazard* event with training data $> {5000}$ patients. These results highlights a strong performance improvement when using deep learning models on non-proportional events, moreover, the benefit of the Transformer compared to the DeepHit model was more pronounced on smaller dataset sizes. Additionally, Fig. 3 shows the mean absolute error of the cause-specific hazard predictions as a function of time. Our Transformer model had better performance on *Proportional hazard* event despite a lower precision at early time steps of this hazards predictions. We observed that our Transformer-based model always had a large benefit towards the end of the simulated time-frame, which indicates a better ability to extrapolate cause-specific hazards from the set of observed events. We also noted that the PyDTS and RCoxPH models had extremely poor performance on the later part of the simulated time where fewer events were observed. This was true for the *Proportional hazard* event, but even more pronounced for non-proportional hazard events.

**Table 1:** Mean absolute error of the cause-specific hazard prediction for datasets of 2000—50,000 patients

N^a^	Event	RCoxPH	PyDTS	DeepHit	Transformer (ours)
2000	Proportional	1.38 (0.83–1.94)	1.27 (0.97–1.57)	**0.98 (0.95–1.00)**	2.16 (1.80–2.53)
	Increasing	3.14 (2.50–3.78)	3.10 (2.55–3.64)	2.10 (2.04–2.17)	**1.56 (1.44–1.68)**
	Non-monotonic	3.74 (2.99–4.49)	3.68 (2.90–4.46)	2.70 (2.65–2.75)	**2.06 (1.93–2.19)**
5000	Proportional	0.88 (0.73–1.04)	0.89 (0.74–1.03)	0.95 (0.91–1.00)	**0.81 (0.71–0.91)**
	Increasing	2.42 (2.09–2.76)	2.42 (2.14–2.70)	2.04 (1.97–2.12)	**1.34 (1.28–1.39)**
	Non-monotonic	3.02 (2.60–3.43)	2.92 (2.57–3.27)	2.68 (2.62–2.73)	**1.70 (1.63–1.76)**
10,000	Proportional	0.71 (0.66–0.76)	0.71 (0.66–0.76)	0.93 (0.86–1.01)	**0.58 (0.55–0.62)**
	Increasing	2.11 (1.71–2.51)	2.09 (1.77–2.40)	2.05 (1.98–2.12)	**1.44 (1.40–1.48)**
	Non-monotonic	2.60 (2.42–2.77)	2.56 (2.39–2.72)	2.54 (2.45–2.64)	**1.73 (1.68–1.78)**
20,000	Proportional	0.58 (0.51–0.65)	0.59 (0.51–0.66)	0.65 (0.62–0.68)	**0.52 (0.50–0.54)**
	Increasing	1.99 (1.83–2.15)	2.02 (1.87–2.17)	1.69 (1.64–1.74)	**1.55 (1.49–1.60)**
	Non-monotonic	2.43 (2.31–2.56)	2.40 (2.28–2.52)	2.17 (2.14–2.21)	**1.84 (1.79–1.88)**
50,000	Proportional	0.54 (0.47–0.60)	**0.53 (0.46–0.60)**	0.61 (0.58–0.63)	0.55 (0.51–0.59)
	Increasing	1.80 (1.47–2.13)	1.82 (1.58–2.05)	1.64 (1.59–1.68)	**1.62 (1.57–1.67)**
	Non-monotonic	2.24 (2.18–2.30)	2.22 (2.16–2.28)	2.12 (2.08–2.15)	**1.83 (1.78–1.88)**

Error was multiplied by 100 for readability. In each line, the best performance is indicated in bold

^a^Number of patients in the simulated dataset

### Evaluation on the ELSA dataset

#### Collected data

The cohort size was 3564 patients. We selected 74 variables of which 54 were binary. Over the 8-year study period, there were 542 diagnoses of *psychiatric conditions*, 150 diagnoses of *dementia*, and 499 recordings of *death*. Cumulative incidences of each event are illustrated in Fig. 2c. The list of selected variables is shown in Table 6 from Appendix. Some variables had a large number of missing values—up to 45%—and 22 variables had more than 10% missing values. The missing values were imputed using the median value for the continuous variables, and the most frequent value for binary variables. Because evaluated models other than the Transformer and RCoxPH models do not inherently support sequential input data, we used singleton-length input data to provide a fair comparison between all models. All models learnt from input singleton-length sequences and produced cause-specific hazard predictions as a fixed-length time series.

#### Performance comparison

Integrated Brier scores and Time-dependent Concordance Index for each model are presented in Table 2. The mean value and 95% confidence interval were obtained by bootstrapping on the test dataset. Our Transformer-based model had the best Integrated Brier Score and Time-dependent Concordance Index. Moreover, the PyDTS model was slighlty better than the RCoxPH model, but in comparison, the Transformer model allowed for a major improvement on both metrics. Finally, despite a strong Integrated Brier Score, the DeepHit model showed a poor Concordance index on the ELSA dataset.

**Table 2:** Integrated brier score and time-dependent concordance index ($C_{td}$ index) for the prediction of three competing events on the English longitudinal study of ageing dataset

	Integrated brier score	*C*_*td*_ index
RCoxPH	0.2385 (0.2334–0.2437)	0.596 (0.5788–0.6133)
PyDTS	0.2402 (0.2352–0.2452)	0.5907 (0.5735–0.6078)
DeepHit	0.2381 (0.2322–0.2440)	0.4232 (0.4075–0.4389)
Transformer	**0.2258** (0.2220–0.2296)	**0.6312** (0.6113–0.6510)

Lower integrated brier score indicates better performance, higher time-dependent concordance index indicates better performance. The table presents the mean metric and 95% confidence interval obtained by bootstrap on the test set. For each metric, the best performance is indicated in bold

### Feature importance

**Fig. 4:**
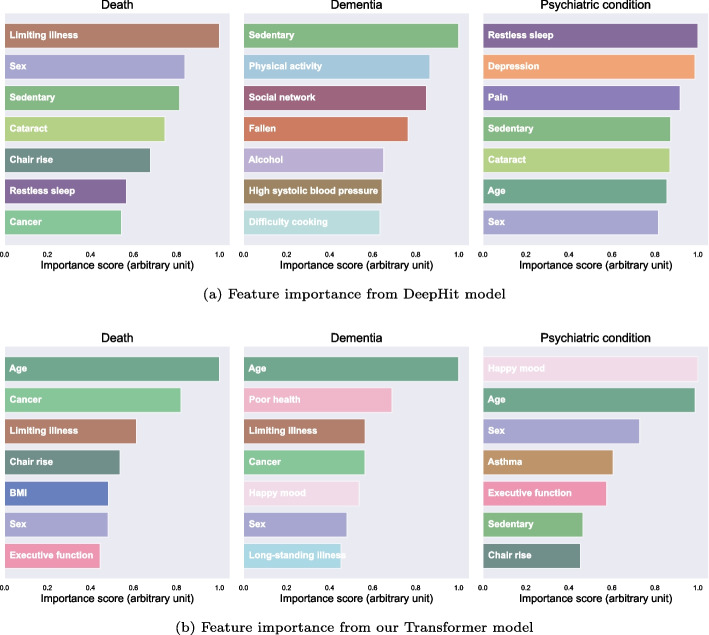
Seven most important features obtained from the mean integrated gradients from the Deephit (**a**) and Transformer (**b**) model using the ELSA dataset

The most important features on average for the prediction of each event by the DeepHit and Transformer models are shown on Fig. 4. See Table 6 from Appendix for details on each feature. The *age* feature was the most important feature for the Transformer model’s predictions. In the prediction of *death*, the Transformer model notably used the binary features *limiting illness* and *cancer*, which stated, respectively, ”Whether limited by longtime illness” and ”Ever diagnosed with cancer”. In the Transformer model predictions, *happy mood* only appeared among the important features of *psychiatric condition* and *dementia* predictions.

### Proportional hazard assumption

Variables that broke the proportional hazard assumption are shown in Table 3. This table lists the variables of each dataset where Schoenfeld residuals of the fitted RCoxPH model had *p* values lower than 0.05. In the synthetic dataset none of the five variables broke the proportional hazard assumption for the *Proportional hazard* event, whereas the *Increasing hazard* event and *Non-monotonic hazard* event had respectively five and four variables breaking the proportional hazard assumption. Events from the ELSA dataset had four to six Schoenfeld residuals with *p* values lower than 0.05. This indicates that the *Death*, *Psychiatric condition*, and *Dementia* events had non-proportional hazard rates.

**Table 3:** Variables from the English longitudinal study of ageing and synthetic datasets for which the *p* value of the Schoenfeld residual from the RCoxPH model was lower than 0.05

Dataset	Event	Variable	*p* value
English longitudinal study of ageing	Death	Lung function	0.015
		Depression	0.023
		Difficulty with money	0.001
		Sex	0.018
	Psychiatric condition	HbA1c	0.036
		LDL	0.043
		Hip fracture	0.005
		Poor hearing	0.019
		Total cholesterol	0.050
	Dementia	Fibrinogen	0.002
		HDL	0.002
		BMI	0.006
		Cataract	0.007
		Difficulty using map	0.017
		Retinopathy	0.003
Synthetic dataset	Proportional	Ø	Ø
	Increasing	Z1	< 0.001
		Z2	< 0.001
		Z3	< 0.001
		Z4	< 0.001
		Z5	< 0.001
	Non-monotonic	Z1	< 0.001
		Z2	< 0.001
		Z3	< 0.001
		Z5	0.004

A *p* value lower than 0.05 for a variable indicates a violation of the proportional hazards assumption

## Discussion

We introduced a Transformer-based deep learning model for the prediction of cause-specific hazards in the context of discrete-time competing risks. This model provides state-of-the-art hazard prediction without strong assumption on the relation between covariates and cause-specific hazard. It strongly outperformed current models even with relatively small training datasets, and was especially successful on events with highly non-proportional hazards or few observed outcomes. We noted that basic models could perform better in a simplistic setting of time-independent proportional hazard with a small training sample; however our Transformer model was generally the best for proportional hazards too.

Our Transformer-based model had the best predictive performance of the cause-specific hazard for sizes simulated datasets ranging from 5000 to 50,000. It also had the best Integrated Brier score and Time-dependent Concordance index on the prediction of three competing events from the ELSA dataset. The experiment on simulated data showed that our model notably outperformed other models in predicting the cause-specific hazards at later time steps where fewer outcomes were observed. This resulted in improved performance on the hazard prediction of rare events, a key benefit of our model. Such behaviour could be expected because of the ability of the Transformer architecture to learn and extrapolate complex temporal features from input data and generate coherent time-series.

The analysis of the proportional hazard assumption on the synthetic data showed that only the *Proportional hazard* event had a proportional hazard rate. This was consistent with the definition of each event. The same analysis on the ELSA dataset indicated that all three events had non-proportional hazards, which is consistent with other findings of departure from the proportional hazard assumption in clinical data [12–15]. As a result, in both the synthetic and ELSA datasets, our model strongly outperformed current models on all events featuring non-proportional hazard rates.

Moreover, our model outperformed the DeepHit model on non-proportional hazard by a larger margin for synthetic datasets with sample sizes of 2000–10,000. This indicates that the Transformer model has a better generalization from limited data. Such results greatly increase the usability of our model on relatively small datasets such as ELSA and most longitudinal cohorts. Additionally, the interpretability through integrated gradients provided the main features that affected the result of a prediction. This can be used by clinicians to ensure trust in the model’s prediction, and focus their attention on features that it deemed most relevant. This is critical for clinical use of any machine learning model as no decision-making ought to be based on a non-explainable prediction.

Some limitations remain in our study. Firstly, our model has a large number of parameters unlike the RCoxPH and PyDTS models. While non-optimized parameters already outperform other models, fine-tuning the network size and training parameters could improve performance. Secondly, our Transformer-based model was consistently better than the simpler architecture of the DeepHit model. However, the gain in performance came with a higher computational cost. This was not limiting in our study as the training times did not exceed several minutes. Finally, to provide a fair comparison between models, only singleton-length input sequences were utilized in the data examples, as models other than the RCoxPH and Transformer were not designed for handling sequential input. This experiment did demonstrate the ability of the Transformer model to generate meaningful sequences, but did not take benefit from its ability to understand complex dynamics of input sequences.

## Conclusions

This study introduces a Transformer-based deep learning model with state-of-the-art performance on the cause-specific hazard prediction in the context of discrete-time competing risks. Our model outperformed current models in cause-specific hazard prediction especially for non-proportional hazard rates and few observed outcomes. It had an increased benefit compared to current models for datasets of 2000–50,000 patients. The designs where our model shows greater benefits encompass those of most clinical survival analysis studies on longitudinal cohorts. Our Transformer-based model is ready to be used for improving current hazard predictions on longitudinal cohorts with complex covariate-to-outcome dynamics.

## Figures and Tables

**Fig. 5:**
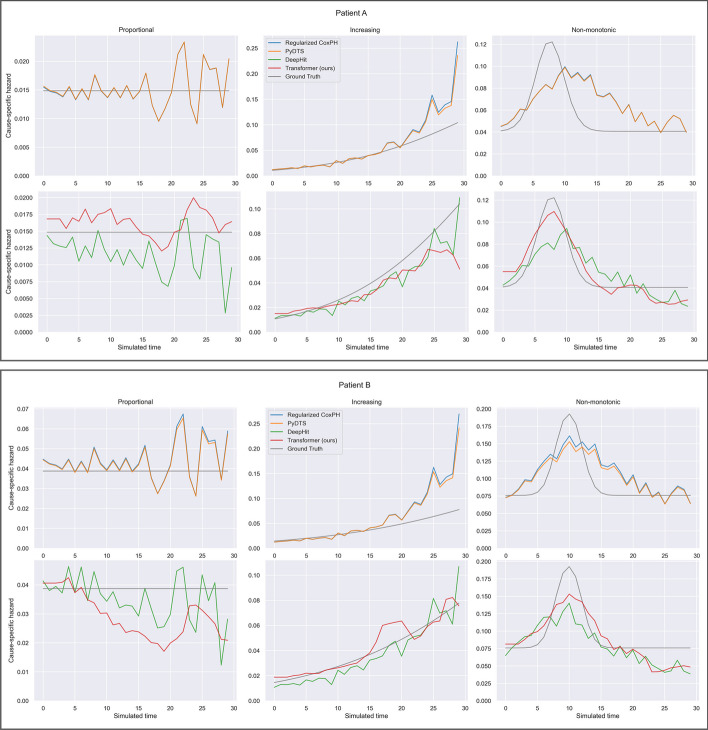
Cause-specific hazard predictions on two patients from the synthetic dataset. The ground truth and predicted hazard are presented for each of the *Proportional*, *Increasing*, and *Non-Monotonic* hazard events. For readability, the PyDTS and RCoxPH models are presented on the top row and the DeepHit and Transformer models on the bottom row

## Data Availability

Our codes and simulated data are openly available at https://github.com/USM-CHU-FGuyon/cause_specific_hazard_transformer

## Appendix 1: Introduction to transformer models

Transformers, introduced by Vaswani et al. [24] have become the go-to architecture for sequence-to-sequence tasks. As shown in Fig. 1, input sequences go through the following stack of modules: Embedding, Transformer Encoder, Linear Decoder. This section provides a qualitative explanation, along with a more detailed description of each module.

*Embedding* Embedding adds temporal information to the input sequences. This allows following blocks to process the embedded sequences as a temporal sequence rather than a unordered set of values.

*Transformer encoder* The Transformer Encoder uses the attention mechanism to extract the information relevant to the prediction task. By learning attention scores, it encodes the input sequences into a vector that depends solely on relevant temporal information from the input sequences. Encoding this vector provides a lower-dimension representation of the input sequences that is easier to process for the prediction task.

*Linear decoder* Vectors encoded by the Transformer Encoder can be decoded into the final prediction using a linear network. This is a simple architecture that processes the input vector using a set of trained weights in a single neuron layer.

### Appendix 1.1: Embedding

Contrary to other recurrent neural networks, the Transformer architecture do not inherently understand temporality of input sequences. The aim of the embedding step is to learn a representation of the input sequences that contains temporal information [32]. The following operations are applied:

1. Input sequences *X* are embedded using a feed-forward network: we denote this embedding $X_{emb} = XF_1^*$ where $F_1^*$ denotes the trained weights for input sequences embedding. This embedding is a representation of the input vector in a slightly lower dimension.
2. A time embedding is then concatenated to the embedded time series $X_{emb}$:
  $$\begin{aligned}&T_{emb} = TF_{time} \\&X_{time - emb} = X_{emb} \oplus T_{emb} \end{aligned}$$
  where *T* are the timesteps of the input sequences *X*, $F_{time}$ is the operator of time-embedding, and $\oplus$ denotes concatenation. We call *time-embedded sequences* the tensor $X_{time - emb}$.
3. Positional encoding is applied then summed to the time-embedded sequences. In Transformer models, the positional encoding operator (PE) is usually defined as such:
  $$\begin{aligned} PE(i, pos) = \sin \left( \frac{pos}{10{,}000^{i/d}}\right) \quad {\text{when}}\; i\; {\text{is even,}}\\ PE(i, pos) = \cos \left( \frac{pos}{10{,}000^{i/d}}\right) \quad {\text{when}}\; i\; {\text{is odd,}} \end{aligned}$$
  where *i* is the index of the time series, *pos* is the position of the element, and *d* the dimensionality of the embedding. This positional encoding operator is applied on the first axis of $X_{time- emb}$, i.e. identically for all patients. It produces a tensor of same shape as the input embedding. The positionnaly-encoded embedding $X_{pe}$ of input sequences *X* is
  $$\begin{aligned} X_{pe} = {{\,\textrm{tanh}\,}}(X_{time - emb})(1+PE) \end{aligned}$$
  The reason for summing the positional encoding to the time-embedded sequences is to preserve the dimensionality of the embedded space, while adding the temporal information to the sequence.

The positionally-encoded sequences $X_{pe}$ are a representation of the input sequences, that include temporal information about the timesteps of measure of the input variables. This tensor is the input of the Transformer encoder. In the following $X_{pe}$ is called the *embedding of X*.

### Appendix 1.2: Transformer encoder

The Transformer encoder is the crux of the Transformer architecture. It features a multi-head attention module followed by layer normalization and a linear layer.

In this work, we used a number of attention heads $n_{head} = 1$ and an embedding dimension $n_{lat} = 64$. A single attention head *h* contains three sub-networks $Q_h$, $K_h$, and $V_h$ respectively called Query, Key, and Value subnetwork. Their respective trained weights are denoted $Q^*_h$, $K^*_h$, and $V^*_h$. An attention head *h* computes the attention of each element *x* using its embedding $x_{pe}$ and the embedding $X_{pe}$ of the input sequences *X*.

1. The embedding of *x* is fed to the Query subnetwork which outputs $q_{x,h} = x_{pe}Q^*_h$
2. The embedding of the input sequences *X* is fed to both the Key and Value subnetworks, which respectively output $k_{X,h} = X_eK^*_h$, and $v_{X,h} = X_eV^*_h$
3. The attention score of the element is given by
  $$\begin{aligned} a_{x,X,h} = q_{x,h}* k_{X,h} \end{aligned}$$
4. The element’s attention output $A_{x,X,h}$ is obtained by weighting $v_{X,h}$ with a function of the attention score $a_{x,X,h}$:
  $$\begin{aligned} A_{x,X,h} = {{\,\textrm{softmax}\,}}\left( \frac{a_{x,h}}{\sqrt{n_{dim}}}\right) * v_{X,h} \end{aligned}$$

The output of the multi-head attention module is a weighted sum of each head’s attention: $A_{x,X} = \Sigma A_{x,X,h}w_h$ where $W = [w_1, \dots w_h]$ is a trained parameter.

The concatenation of all elements’ attention yields the attention matrix:

$$\begin{aligned} A_X = \bigoplus _{x \le {n_{var}+1}} A_{x,X} \end{aligned}$$

Attention captures complex relationships between a number of input sequences. It weights the informativeness of each input sequence within the context of the whole input sequences. The subsequent normalization and feed forward networks use the attention matrix to produce a lower dimension latent representation of the input sequences. Weakly-informative elements of the input sequences, eg. highly correlated other input sequences, will obtain a low attention value and will scarcely contribute to the latent representation.

Attention and embeddings of the input sequences are then given to a feed-forward encoder to produce the final latent representation $X_l$.

In short, this attention mechanism allows generating a latent representation of large and complex input sequences by effectively compressing embeddings of the input sequences in a way that preserves informative values and their temporality.

### Appendix 1.3: Linear decoder

The feed-forward decoder uses the latent representation $X_l$ for the prediction task at hand. The predicted values are $P = X_lF_{dec}^*$ where $F_{dec}^*$ are the trained weights of the decoder. In this encoder–decoder architecture, modules learn in unison to respectively encode the large input data to a relevant latent space and to utilize the latent representation for producing accurate predictions.

This architecture is able to process a large amount of input data while keeping reasonable dimensionality of the training weights. This is especially helpful to improve computation times and reduce the risk of overfitting.

### Appendix 1.4: Implicit assumptions

The Transformer architecture allows to make prediction without explicit assumptions on the predicted variable. Its effcience has been shown experimentally in multiple fields of application. However, it features some implicit assumptions that should be stated. *Positional encoding effectively conveys temporal information to the model* This architecture assumes that the use of sinusoidal functions is efficient for conveying the temporal information to the Transformer encoder. This was not rigorously demonstrated but this method’s effectiveness was empirically observed. Nevertheless, positional encoding could fail to capture some nuances of temporal dependency.

*Attention is stationary* The attention mechanism does not explicitly compute a temporal variation of the variable informativeness. This can be problematic if a series of a variable contains highly informative values at some times, and non-informative values the rest of the time. However, the initial embedding may isolate such highly informative values and mitigate the limitations caused by this assumption.

*Attention as a proxy for relevance* Attention as computed by the multi-head attention module is based on learning parameters that identify relations between a set of input and output sequences. This concept might not perfectly align with human notion of relevance.

## Appendix 2: Additional results

### Appendix 2.1:Individual prediction visualization

We presented some individual patients’ predicted hazards on Fig. 5. This figure illustrates the ability of the Transformer model to produce meaningful and individualized predictions, which greatly improves usability in clinical practice. The RCoxPH and PyDTS model offer decent average performance but fail to produce individually accurate hazard estimates.

### Appendix 2.2: Peak hazard time prediction

Using the *Non-monotonic hazard* event, we designed an experiment to evaluate each model’s ability to create individualized predictions. This is not a standard metric but rather a qualitative insight of models’ performance. The *Non-monotonic hazard* event reaches a maximum hazard value between the 3rd and 25th time steps. We compared the time at maximum hazard between the ground-truth and predicted values. The mean absolute error is presented in Table 4. We observed that the Transformer model achieves a much better performance, highlighting its ability to produce a meaning temporal prediction for each patient rather than predictions that are only good *on average*.

**Table 4:** Mean absolute error in predicting the time of maximum hazard for the simulated *Non-monotonic hazard* event

	Mean absolute error
model	
RCoxPH	3.5
PyDTS	3.5
DeepHit	2.9
Transformer (ours)	1.7

The time of maximum hazard ranged from time step 3 to time step 25. RCoxPH: Regularized Cox Proportional Hazards

## Appendix 3: Supplementary tables

See Tables 5 and 6.

**Table 5:** Definition of the cause-specific hazard $\lambda _X(t)$ for each event of the simulation

	Proportional hazard	Increasing hazard	Non-monotonic hazard
$\lambda _X(t)$	$a \cdot {{\,\textrm{expit}\,}}(f(X,t) + X^T \beta )$		
*f*(*X*, *t*)	$-0.95 \cdot X^T \alpha$	$-1 + 0.1 \ t \cdot X^T \alpha$	$10\sigma \cdot \exp (\frac{(t-\mu (X))^2}{2\sigma (X)^2})$
$\alpha$	[0.25, 1, 0.25, 0.25, 0.25]		Ø
$\beta$	$[0, -1.1, -1.4, -1.1, -0.7]$	$[-1.6, -1.1, -1.4, -1.1, -0.7]$	$[0.22, -1.1, -1.1, -0.9, -0.7]$
$\mu (X)$	Ø		$X^T[12,6,4,2,0]$
$\sigma (X)$	Ø		$X^T [1.2,2.5,0.8,1,0.2]$

X denotes a vector of five covariates uniformly distributed between 0 and 1

**Table 6:** Retained variables from the ELSA dataset

Feature	Definition from the ELSA dataset	Data type	Imputation rate (%)
*General*			
Age	Age (years)	Continuous	0
Sex	Sex	Binary	0
Waist	Waist circumference (cm)	Continuous	26
BMI	Body mass index (kg/m^2^)	Continuous	29
Marital status	Whether married	Binary	0
Living alone	Whether lives alone	Binary	0
Sedentary	Physical activity summary: sedentary	Binary	2
Sport	Whether practices weekly vigorous physical activity	Binary	2
Tobacco	Whether respondent smokes every day	Binary	0
Alcohol	Alcohol consumption 6+ days a week	Binary	3
*Lab*			
Apo E	Blood apolipoprotein E level (mol/L)	Continuous	44
Ferritin	Blood ferritin level (ng/mL)	Continuous	44
Fibrinogen	Blood fibrinogen level (g/L)	Continuous	44
HbA1c	Blood glycated haemoglobin level (%)	Continuous	45
HDL cholesterol	Blood high-density lipoprotein level (mol/L)	Continuous	44
LDL cholesterol	Blood low-density lipoprotein level (mol/L)	Continuous	45
Total cholesterol	Total cholesterol (mol/L)	Continuous	44
Pulse Pressure	Valid pulse pressure (mmHg)	Continuous	33
Systolic blood pressure	Measured diastolic blood pressure (mmHg)	Continuous	33
Diastolic blood pressure	Measured diastolic blood pressure (mmHg)	Continuous	33
High blood pressure	Measured systolic blood pressure $\ge$ 140 mmHg	Binary	33
Best lung function	Highest forced expiratory volume reading (L)	Continuous	31
Mean lung function	Mean pulmonary function readings (L/min)	Continuous	30
Grip strength	Maximum grip strength (kg)	Continuous	24
*Medications*			
Blood pressure medication	Whether taking medication for high blood pressure	Binary	27
Asthma medication	Whether taking medication for asthma	Binary	0
Beta-blockers	Whether taking beta-blocker	Binary	10
*Diagnoses*			
Lung disease	Ever diagnosed with lung disease	Binary	1
Asthma	Ever diagnosed with asthma	Binary	1
Arthritis	Ever diagnosed with arthritis	Binary	1
Cancer	Ever diagnosed with cancer	Binary	1
Cardiovascular	Ever diagnosed with Infarction, stroke or heart failure	Binary	1
Cataract	Ever diagnosed with cataract	Binary	0
Diabetes	Ever diagnosed with diabetes	Binary	1
Heart attack	Ever diagnosed with myocardial infarction	Binary	1
Retinopathy	Ever diagnosed with retinopathy	Binary	0
High blood pressure	Ever diagnosed with high blood pressure	Binary	1
Stroke	Ever diagnosed with stroke	Binary	1
Glaucoma	Ever diagnosed with glaucoma	Binary	0
Macular	Newly diagnosed macular degeneration	Binary	0
*Self-reports*			
Poor hearing	Whether reported poor hearing	Binary	0
Poor vision	Whether reported poor vision	Binary	0
Poor health	Whether reported poor health	Binary	2
Difficulty cooking	Difficulty preparing a hot meal	Binary	
Difficulty taking medications	Difficulty taking medications	Binary	0
Difficulty using map	Difficulty using a map	Binary	0
Difficulty with money	Difficulty managing money	Binary	0
Limited by illness	Whether limited by longtime illness	Binary	0
Restless sleep	Whether felt their sleep was restless during the past week	Binary	3
Breathlessness	Respiratory questionnaire : indication of breathlessness	Binary	14
Dizziness	Frequency of dizziness when walking on level surface	6 values	0
Mobility difficulties	Difficulty getting up from chair after sitting long periods	Binary	0
Exhaustion	Whether respondent felt everything they did during the past week was an effort	Binary	3
Falls	Whether ever fallen down	Binary	0
Social network	Whether the respondent has any friends	Binary	0
Happy mood	Whether respondent was happy much of the time during the past week	Binary	18
No relay	Whether the respondent did not have any friends or responded they could not rely on their friends	Binary	18
Pain	Whether often troubled with pain	Binary	2
Debt	Any money owed to friends, relatives, or other private individuals	Binary	1
Hip fracture	Whether had fractured hip or joint replacement	Binary	0
Incontinence	Whether lost urine beyond control in last 12 months	Binary	2
Long-standing illness	Whether reported a long-standing illness	Binary	0
Heating: coal	Whether uses coal for heating	Binary	0
Heating: electricity	Whether uses electricity for heating	Binary	0
Heating: gas	Whether uses gas for heating	Binary	0
Heating: oil	Whether uses oil for heating	Binary	0
Heating: other	Whether uses other fuel for heating	Binary	0
Heating: paraffin	Whether uses paraffin for heating	Binary	0
Heating: wood	Whether uses wood for heating	Binary	0
*Tests*			
Depression scale	Whether respondent had $\ge 4$ out of 8-item of the Center for Epidemiologic Studies depression scale	Binary	2
Letter search	Accuracy at letter search test ($\%$)	Continuous	7
Executive function	Executive function index	20 values	7
Memory function	Memory function index	28 values	3
Chair rise	Whether successfully stood up from chair	Binary	10

## Abbreviations

ELSA: English longitudinal study of ageing
LSTM: Long short-term memory
RCoxPH: Regularized Cox proportional hazards model
